# Maximizing limited space: usefulness of percutaneous cricothyrotomy and super-soft hood for hypopharyngeal endoscopic submucosal dissection

**DOI:** 10.1016/j.vgie.2023.06.009

**Published:** 2023-08-18

**Authors:** Yuto Shimamura, Marc Julius Navarro, Yohei Nishikawa, Mai Fukuda, Yoshiaki Kimoto, Takashi Suzuki, Haruhiro Inoue

**Affiliations:** 1 Digestive Diseases Center, Showa University Koto Toyosu Hospital, Tokyo, Japan; 2 Digestive Diseases Center, Showa University Koto Toyosu Hospital, Tokyo, Japan; 3 Institute of Digestive and Liver Diseases, St. Luke’s Medical Center, Quezon City, Philippines; 4 Digestive Diseases Center, Showa University Koto Toyosu Hospital, Tokyo, Japan; 5 Department of Anesthesiology, Showa University Koto Toyosu Hospital, Tokyo, Japan; 6 Digestive Diseases Center, Showa University Koto Toyosu Hospital, Tokyo, Japan

## Abstract

Video 1 Demonstration of the usefulness of percutaneous cricothyrotomy and a super-soft hood for hypopharyngeal endoscopic submucosal dissection.

Demonstration of the usefulness of percutaneous cricothyrotomy and a super-soft hood for hypopharyngeal endoscopic submucosal dissection.

## Background

Recent advances in GI endoscopy enable us to resect superficial hypopharyngeal carcinomas minimally invasively by endoscopic submucosal dissection (ESD) and endoscopic laryngopharyngeal surgery with the patient under general anesthesia (GA).

Lesions for ESD and endoscopic laryngopharyngeal surgery situated in the oropharynx and hypopharynx are usually technically challenging to resect because of the limited space for the endoscope and other devices to maneuver. The endotracheal (ET) tube inserted to protect and secure the airway sometimes impairs endoscopic manipulation.^1^, ^2^, ^3^ To solve this problem, in our institution, if there is no determined contraindication (such as previous tracheal surgery, fractured larynx, laryngotracheal disruption, and pediatric patients), we conduct pharyngeal ESDs with the patient under GA with controlled ventilation through a percutaneous uncuffed small-bore cricothyrotomy tube with balloon occlusion of the subglottic airway performed by the anesthetist. This prevents the ET tube from affecting endoscopic maneuverability and is beneficial for postoperative airway management in the event of potentially life-threatening laryngeal edema.^4^ We also use a super-soft hood—a newly developed transparent distal attachment capable of adjusting its tip to the shape of narrow spaces because of its flexibility.^5^^,^^6^

## Case

**Figure 1:**
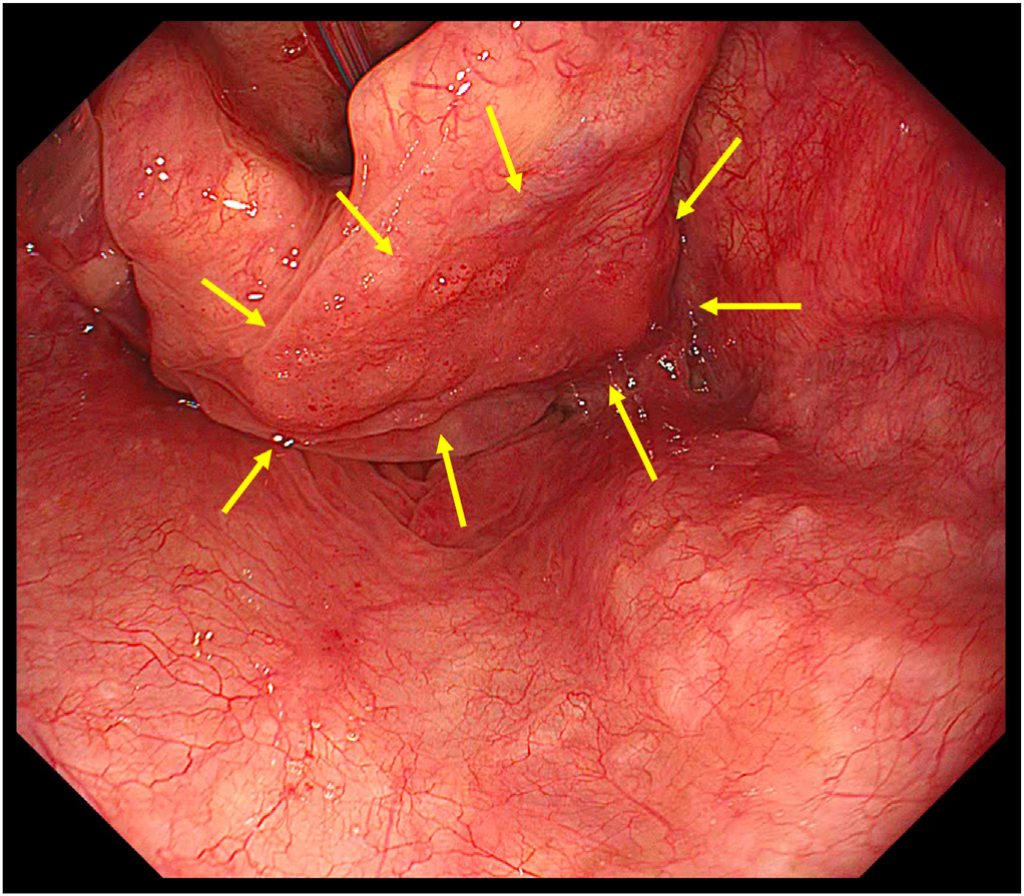
Lesion on white-light endoscopy.

**Figure 2:**
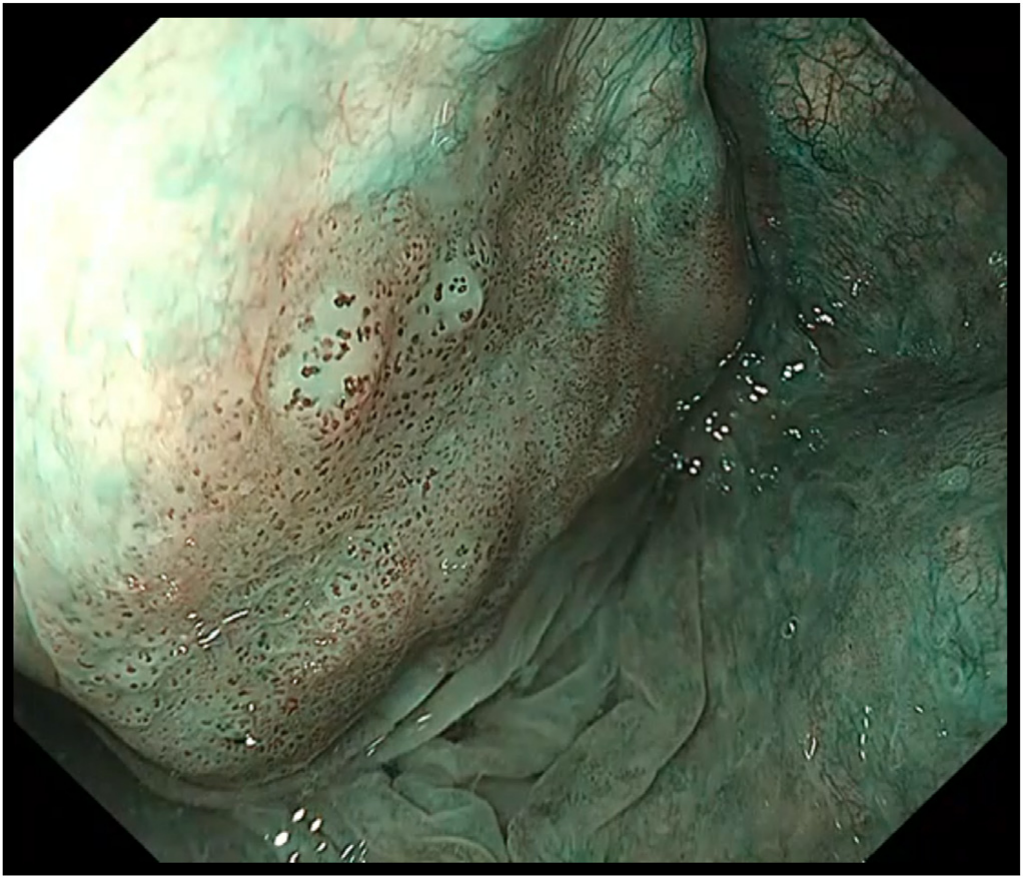
Lesion on magnifying endoscopy with narrow-band imaging.

A 59-year-old man underwent ESD for a superficial hypopharyngeal squamous cell carcinoma (SCC). White-light endoscopy revealed an approximately 20-mm, flat, reddish mucosal irregularity (Paris 0-IIb) at the right piriform sinus of the hypopharynx (Fig. 1). On magnifying endoscopy with narrow-band imaging, the lesion could be recognized as a brownish area with an abnormal intrapapillary capillary loop pattern exhibiting dilation, irregular caliber, and irregular form variation (Type V-1 pattern) (Fig. 2). A biopsy confirmed the diagnosis of hypopharyngeal SCC. A CT scan revealed no lymphadenopathy and distant metastasis.

**Figure 3:**
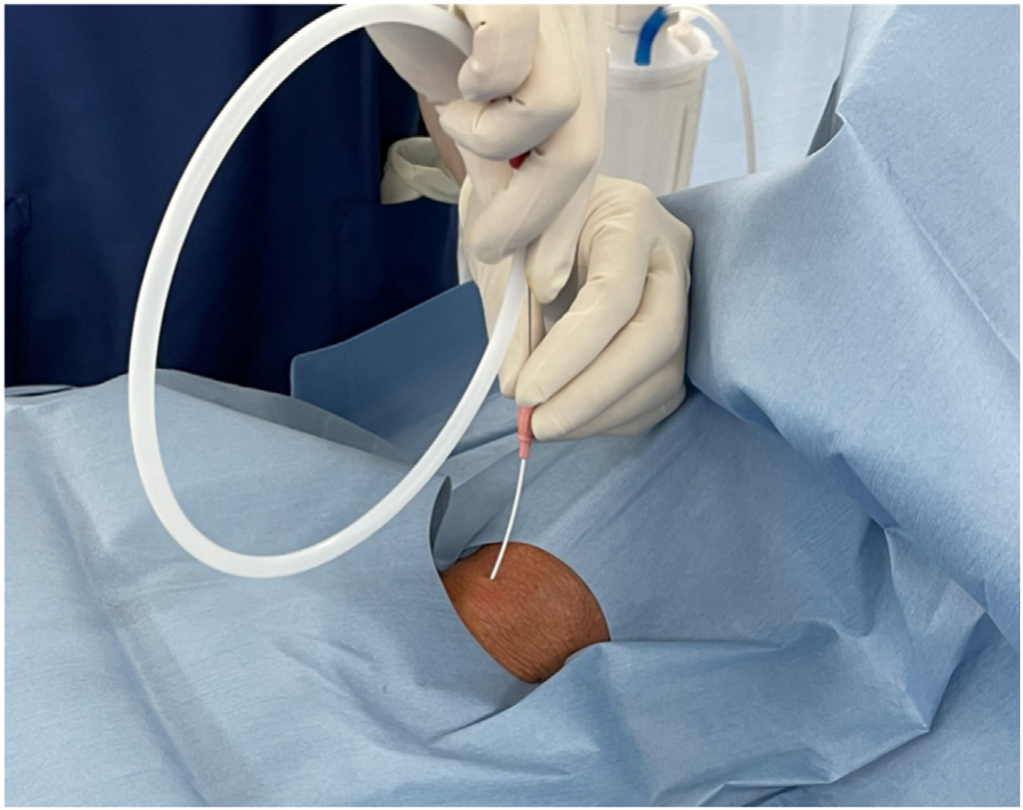
Percutaneous cricothyrotomy.

## Procedure

### Setting of ESD

**Figure 4:**
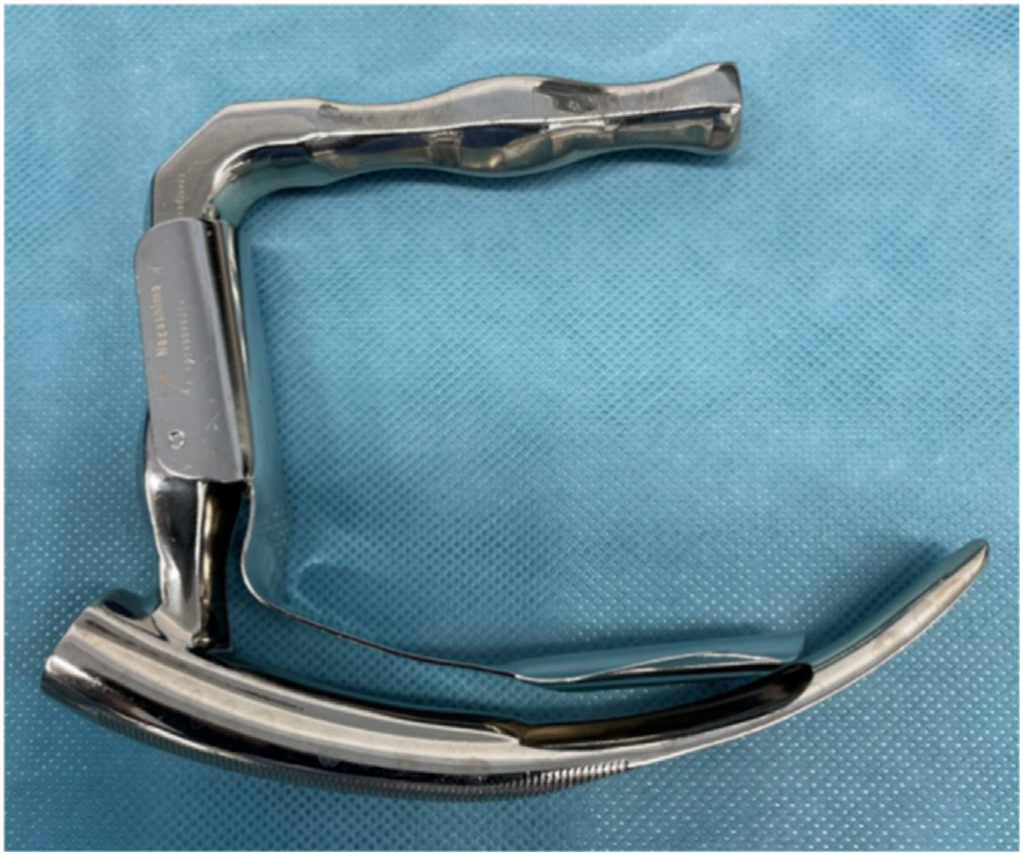
Rigid surgical laryngoscope.

**Figure 5:**
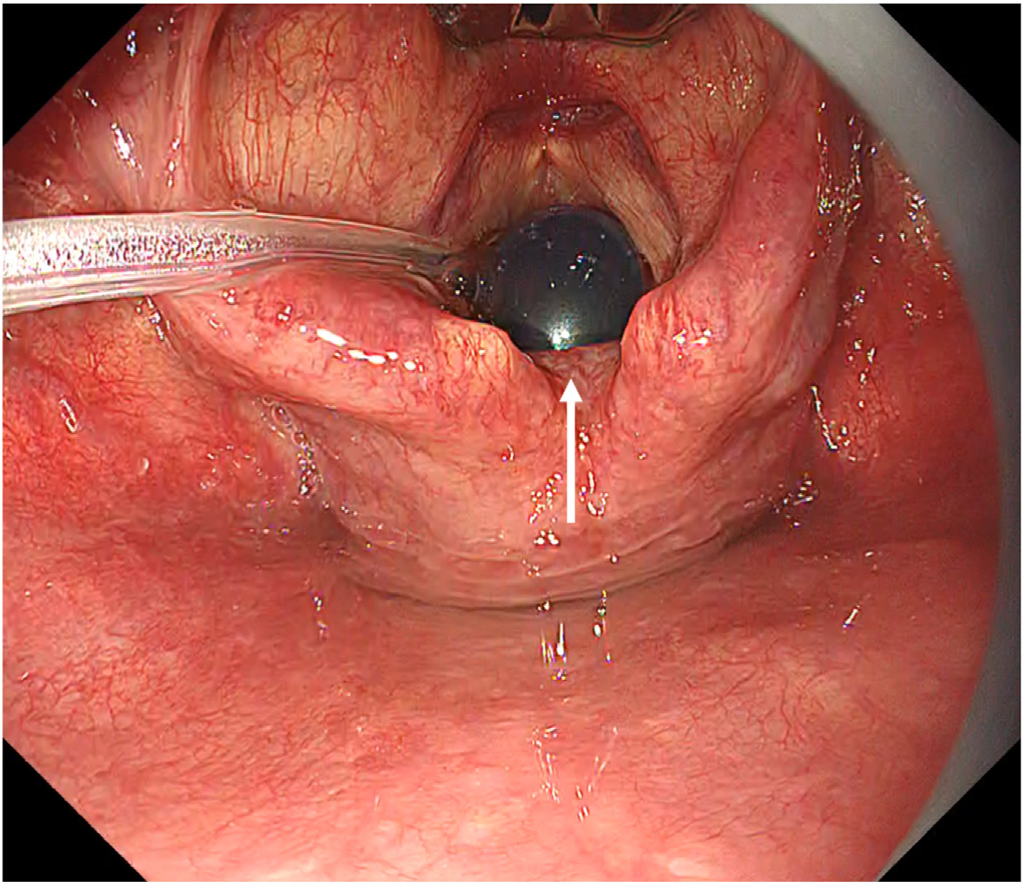
Balloon occlusion of the subglottic airway.

The procedure (Video 1, available online at www.videogie.org) was performed with the patient under GA with controlled ventilation through a percutaneous cricothyrotomy tube. The patient was kept in the supine position. Percutaneous cricothyrotomy using an uncuffed small-bore cricothyrotomy tube (Portex Minitrach II, 4-mm internal diameter; Smiths Medical International Ltd, Luton, United Kingdom; or Melker, 4-mm internal diameter; Cook Medical, Bloomington, Ind, USA) was performed by the anesthetist (Fig. 3). A rigid surgical laryngoscope (Nagashima Medical Instrumental Co, Tokyo, Japan) was inserted to expose the surgical field in the hypopharyngeal area (Fig. 4). Balloon occlusion of the subglottic airway using a bronchial blocker (Coopdech endobronchial blocker with spindle-type cuff; Daikin, Japan) was then inserted orally to avoid air leakage of the controlled ventilation (Fig. 5).

**Figure 6:**
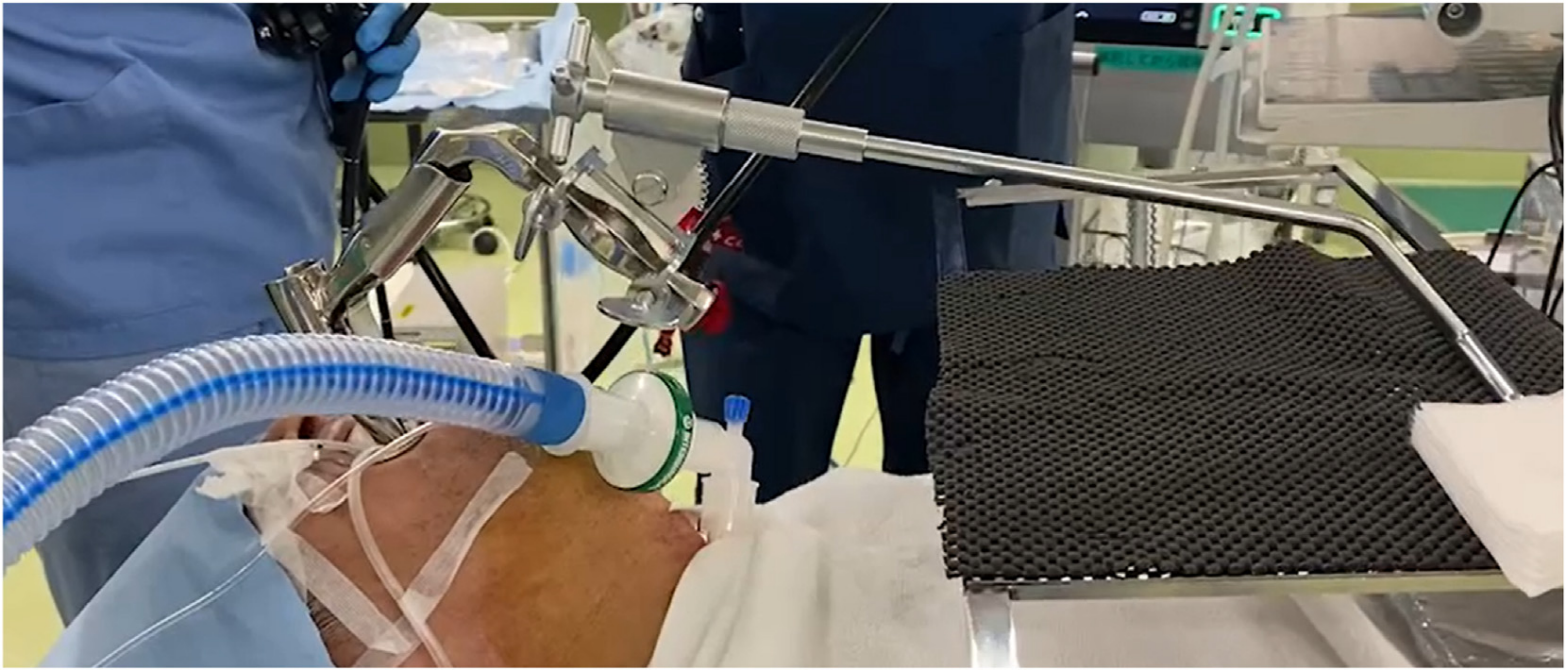
Instrumental setup.

A high-definition magnifying endoscope (GIF-XZ1200; Olympus Corp, Tokyo, Japan) and therapeutic endoscope (GIF-H290T; Olympus Corp) with a super-soft transparent distal attachment (Space Adjuster; TOP Corporation, Tokyo, Japan) were used in the procedure. An electrosurgical knife with water-jet function (ORISE Proknife 2.0 mm; Boston Scientific, Marlborough, Mass, USA) was used for marking, mucosal incision, submucosal injection, submucosal dissection, and hemostasis. An electrogenerator (VIO300D; ERBE, Tübingen, Germany) was also used with the following settings: Marking—Soft Coag, Effect 3, 20W; Cut—Endo Cut I, Effect 2, Duration 2, Interval 2; Coagulation—Swift Coag, Effect 4, 30W.

### ESD Procedure

**Figure 7:**
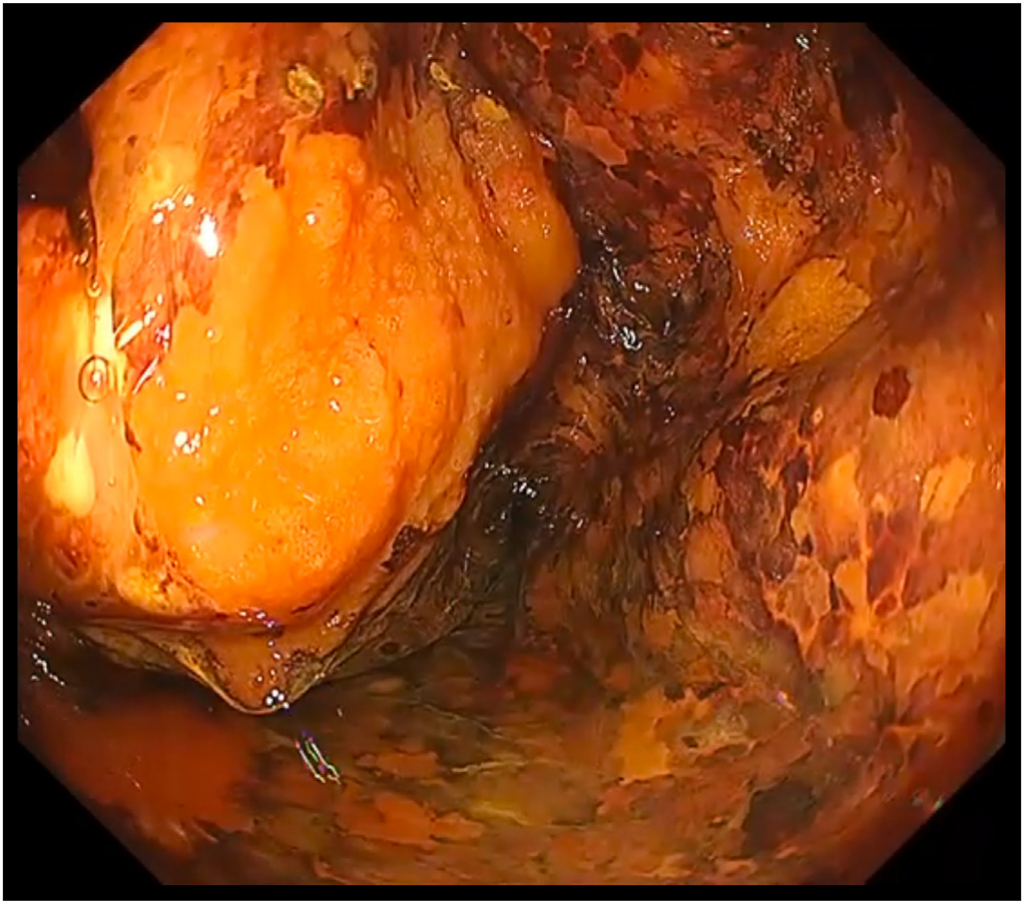
Lesion on chromoendoscopy with 1% iodine solution.

**Figure 8:**
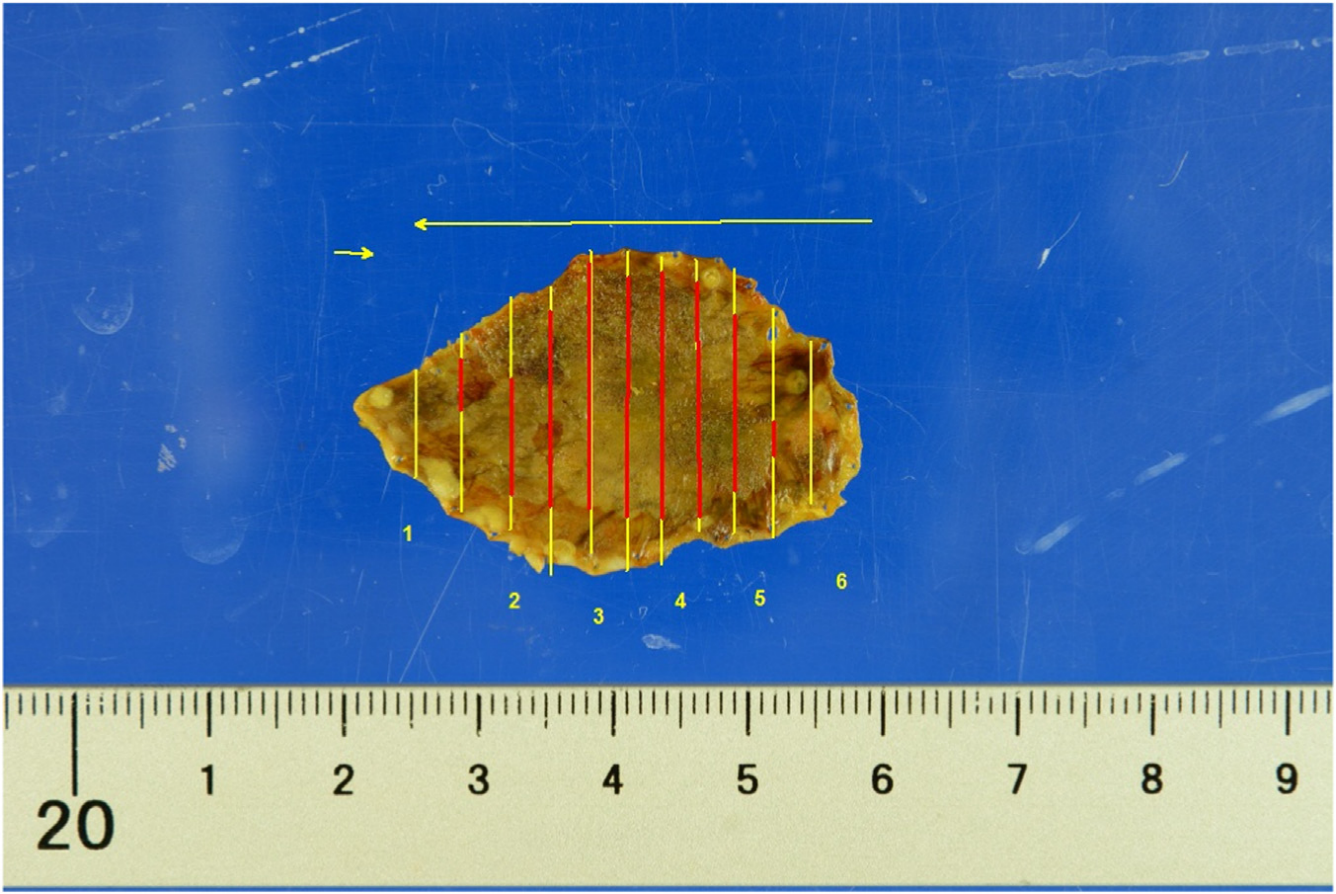
Resected lesion. *Red* and *yellow lines* were tumor margins marked by the pathologist. The *red line* signifies positive area for SCC, and the *yellow line* signifies negative area for malignancy.

After securing the airway and adequately exposing the hypopharyngeal area (Fig. 6), a detailed lesion evaluation was performed. Markings were placed approximately 1 to 2 mm outside the identified lesion margin. Then, we sprayed 1.0% iodine solution using a spraying catheter to confirm the tumor margin and adequacy of the placed markings (Fig. 7). Normal saline with indigo carmine was injected using an injection needle along the markings to create a submucosal bleb. The initial mucosal incision, followed by submucosal dissection, was completed. The lesion was resected en bloc without any adverse event, with a total procedure time of 70 minutes. A total area of 27 × 18 mm was resected, with histopathologic evaluation showing SCC in situ (pTis, tumor thickness 300 um, ly0, v0, pHM0, pVM0) (Fig. 8).

## Outcome

Postoperatively, the percutaneous cricothyrotomy tube was left in situ for suctioning and airway control during possible or actual laryngeal edema until day 1 postprocedure. No notable ESD-related or anesthesia-related adverse events were recognized. Diet was resumed and well-tolerated starting on day 2 postprocedure. The patient was discharged, improved, and stable after 6 hospital days. Six months post-ESD, the patient claimed to be well and asymptomatic. Surveillance EGD showed no evidence of recurrence, and a CT scan revealed unremarkable findings.

This report presents a case of en bloc resection of right piriform sinus SCC via hypopharyngeal ESD. We highlight the usefulness of percutaneous cricothyrotomy and a super-soft hood in maximizing limited space of the pharynx. This case suggests that in more challenging oropharyngeal or hypopharyngeal ESDs, requesting the anesthetist to perform percutaneous cricothyrotomy (instead of ET intubation) for airway management will prevent the ET tube from affecting endoscopic maneuverability in a limited space.

## Disclosure

*Dr Inoue is an advisor at Olympus Corporation and TOP Corporation. All other authors did not disclose any financial relationships.*

## Supplementary data

Video 1 Demonstration of the usefulness of percutaneous cricothyrotomy and a super-soft hood for hypopharyngeal endoscopic submucosal dissection.
